# Understanding Chilling Injury and Sugar Metabolism-Related Genes and Metabolites in ‘Red Haven’ Peaches

**DOI:** 10.3390/plants14142133

**Published:** 2025-07-10

**Authors:** Macarena Farcuh

**Affiliations:** Department of Plant Science and Landscape Architecture, University of Maryland, College Park, MD 20742, USA; mfarcuh@umd.edu

**Keywords:** *Prunus persica* (L.) Batsch, chilling injury, sugar metabolism, gene expression, postharvest

## Abstract

Although cold storage is commonly used to extend peach fruit shelf-life, chilling injury (CI) can develop during low-temperature storage conditions and be expressed during exposure to ambient temperature. Therefore, the objectives of this study were to characterize and compare the differences in CI occurrence as well as sugar metabolism-related genes and metabolites in ‘Red Haven’ peaches stored at 0 °C and 5 °C, followed or not by storage for 3 days (d) at 20 °C (to simulate retail shelf conditions for the evaluation of CI incidence), together with fruit stored at 20 °C, and to identify significant associations between peach CI and sugar metabolism via multivariate analysis. Fruit collected at commercial maturity was stored at 0 °C, 5 °C, and 20 °C and assessed at harvest (0 d) and at 1, 3, 5, 15, and 30 d of storage, followed or not by storage for 3 d at 20 °C. Peaches kept for 30 d at 5 °C plus 3 d at 20 °C exhibited CI, expressed as decreased expressible juice. CI susceptibility was associated with reduced sucrose and increased glucose and fructose, while sorbitol contents were also lower in fruit stored at 5 °C, compared to those stored at 0 °C. This was paralleled by decreased expression of sucrose biosynthesis-related genes and by increased expression of sucrose and sorbitol breakdown-related genes as early as after 5 d of storage at 5 °C. Sugar metabolism changes that occurred during cold storage were maintained after exposure for 3 d to a temperature of 20 °C. The correlations between the evaluated features implied that alterations in sugar metabolism can modulate changes in CI susceptibility. These findings suggest that storage at 0 °C better preserves the sucrose homeostasis of ‘Red Haven’ peaches, reducing CI risk.

## 1. Introduction

Within the Rosaceae family, peaches (*Prunus persica* (L.) Batsch) are considered amongst the most consumed fruits worldwide. However, when kept at room temperature, peaches can rapidly deteriorate. Therefore, cold storage is used to extend the postharvest life of the fruit by slowing down the ripening process [1,2]. Nonetheless, peaches are a chilling-sensitive fruit and are consequently highly vulnerable to the incidence of physiological disorders like chilling injury [3,4]. Fruit exposed to extended low-temperature storage are more prone to developing chilling injury, particularly when stored between 2.2 and 7.6 °C, compared to those stored at 0 °C [5], although the expression of the disorder develops once the fruit is transferred from storage to ambient conditions (20 °C) [6]. Previous studies have shown that 5 °C is considered a chilling injury-inducing temperature (compared to 0 °C) as it triggers important structural changes, particularly in cell wall metabolism, within the fruit, such as the modification of pectin content covalently bound to the cell wall as well as alterations in cell wall-modifying enzyme activities [5]. Chilling injury development negatively impacts fruit quality, increasing fruit losses.

Chilling injury incidence comprises the development of several symptoms, some which are visible and related to alterations in peach fruit texture, such as mealiness and/or leatheriness (lack of juice in the former and lack of softening in the latter), or related to alterations in fruit flesh, such as flesh bleeding and/or flesh browning, and some of which cannot be visually detected, such as flavor loss [5]. In general, the onset of chilling injury commences with flavor loss, followed by the expression of mealiness, whereas the final symptom to appear corresponds to flesh browning [6,7,8]. The visible symptom of mealiness can be assessed based on the expressible juice content, as the latter negatively correlates with the incidence of mealiness in the fruit [9]. The visually undetectable symptom of flavor loss results from alterations in the components of fruit flavor, which include sugars (sweetness of the fruit), acids (acidity of the fruit), and volatile compounds emitted by the fruit (fruit aroma) [2,10,11]. Previous work characterized changes in aroma volatile profiles during the postharvest storage of ‘Red Haven’ peaches with different levels of chilling injury incidence, and 13 key aroma volatiles were identified that may serve as early indicators of chilling injury onset [2]. Nevertheless, research is still lacking on how changes in other fruit flavor components, i.e., sugars, contribute to the development of the symptom of flavor loss, and thereby to chilling injury incidence, in ‘Red Haven’ peaches during cold storage, but particularly after exposure to simulated retail shelf conditions (3 d at 20 °C).

Sugars, in addition to supplying energy and carbon to fruits, also play crucial roles in fruit flavor, as fruit sweetness is primarily defined by the type of sugars present in the fruit as well as their concentration [12,13,14]. Furthermore, sugars have been reported to be involved in cold tolerance during low-temperature exposure in fruit [15,16,17,18,19,20], mainly due to their ability to work as cryoprotectants and function as cell membrane stabilizers and osmoregulators, as well as their antioxidant capacity [20,21,22,23,24,25,26]. In peaches, sucrose (Suc) and sorbitol (Sor) are the main sugars translocated to the fruit, but Suc has been reported to be the prevalent sugar in ripe fruit, while glucose (Glu), fructose (Fru) and Sor are present in lower concentrations [1,27]. Previous studies in peaches have associated higher Suc concentrations in fruit assessed immediately after cold storage (i.e., without subsequent exposure for 3 d to a temperature of 20 °C) with lower chilling injury incidence [16], while others have indicated that increased Suc, Glu, and Fru concentrations are necessary for enhanced chilling injury tolerance [28,29]. In other cases, only increased concentrations of Glu and Fru during cold storage have been shown to increase chilling tolerance, as, for example, in other Rosaceae family members such as apricots [30] and loquats [15]. Moreover, a lack of Sor accumulation has been linked to increased chilling injury incidence in peaches [1,16,29], although its importance relative to Suc is still unclear. To add more complexity to the matter, information on the impact of different storage temperatures, followed or not by subsequent simulated retail shelf conditions (3 d at 20 °C), on peach fruit sugar concentrations and their relationship with chilling injury incidence is limited.

Sugar metabolism is regulated by different enzymes that can also play important roles in chilling injury tolerance in fruits. The synthesis of Suc results from the activity of sucrose phosphate synthase (SPS), using Glu and Fru as substrates [31]. The breakdown of Suc results from the activity of sucrose synthase (SS), which produces Fru and uridine diphosphate glucose (UDP)-Glu [32], as well as from the action of invertases, catabolizing Suc into Glu and Fru [33]. Depending on their optimal pH, invertases can be differentiated into acid or neutral, with the former being reported to be key in Suc metabolism due to its impact on stress responses [34,35]. Within acid invertases, it has been shown that in peaches, only the expression of vacuolar invertases (*PpVIN2*) presents sensitivity to chilling temperatures [36]. Furthermore, invertases, and particularly vacuolar invertases, can be regulated by invertase inhibitors (INH) [33]. Moreover, in the case of Sor, sorbitol dehydrogenase (SDH), which is responsible for the breakdown of Sor into Fru in the presence of nicotinamide adenine dinucleotide (NAD^+^), is the main enzyme responsible for Sor metabolism [37]. As a result of the importance of all of these enzymes in the control of peach sugar metabolism, it is crucial to understand their associations with chilling injury development under different postharvest storage temperatures.

Based on this context, the hypothesis of this work was that different postharvest storage temperatures alter sugar metabolism and therefore impact ‘Red Haven’ peach chilling injury susceptibility. The objective of this study was two-fold: first, to characterize and compare the differences in chilling injury occurrence as well as in sugar metabolism-related genes and metabolites in ‘Red Haven’ peach fruit exposed to temperatures of 0 °C and 5 °C during postharvest storage, followed or not by simulated retail shelf conditions (3 d at 20 °C), alongside fruit stored at 20 °C, and second, to detect significant associations between chilling injury and sugar metabolism via multivariate analysis in peaches.

## 2. Results

### 2.1. Chilling Injury Incidence

**Figure 1:**
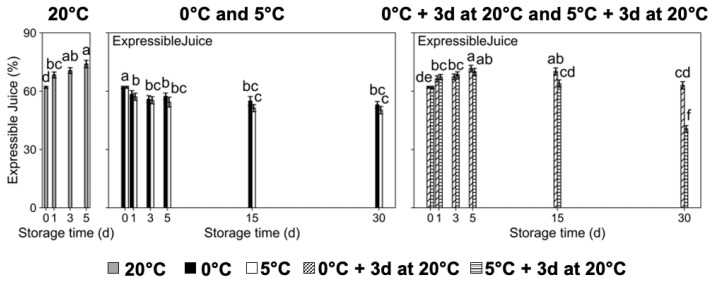
Expressible juice contents of ‘Red Haven’ peaches during storage at different temperatures (20 °C, 0 °C, 5 °C, and 0 °C plus 3 d at 20 °C, and 5 °C plus 3 d at 20 °C). Values are means ± SE (*n* = 4). Distinct letters within each graph represent statistically significant differences (*p* ≤ 0.05). Days (d).

An assessment of expressible juice was conducted to measure the development of the visible symptom of mealiness, and therefore objectively determine chilling injury incidence (Figure 1). Fruit stored at 20 °C displayed a significant increase in expressible juice contents during storage, reaching values ~74% at 5 d (Figure 1). Due to excessive fruit flesh softening, fruit stored at 20 °C could not be assessed after 5 d of storage [2]. Fruit at cold temperatures of 0 °C or 5 °C, and directly assessed afterwards, presented, throughout all postharvest timepoints, expressible juice contents that were significantly lower than those recorded at harvest (Figure 1). Significantly lower expressible juice contents were observed for peaches stored at 5 °C plus 3 d at 20 °C (to simulate retail shelf conditions for the evaluation of CI incidence) compared to fruit stored at 0 °C plus 3 d at 20 °C, 15 d and 30 d postharvest (Figure 1). The highest expression of chilling injury was detected in fruit stored for 30 d at 5 °C plus 3 d at 20 °C, as these fruits presented the lowest expressible juice contents (<40%). No other visible CI symptoms, such as flesh bleeding or browning, were observed in this work. The lack of browning could be attributed to ‘Red Haven’s’ overall browning potential, as it has been shown that the browning potential of peaches depends on the total amount of phenolic compounds present in the fruit as well as on the level of activity of polyphenol oxidase [5].

### 2.2. Sugar Concentration

**Figure 2:**
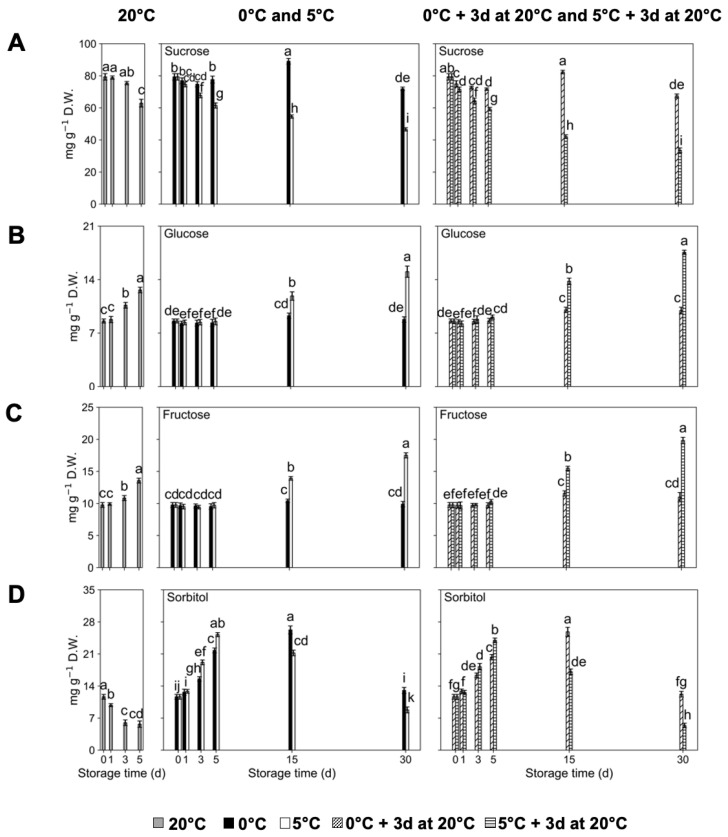
Sugar concentration changes in ‘Red Haven’ peaches during storage at different temperatures (20 °C, 0 °C, 5 °C, and 0 °C plus 3 d at 20 °C, and 5 °C plus 3 d at 20 °C). (**A**) Sucrose; (**B**) glucose; (**C**) fructose; (**D**) sorbitol. Values are means ± SE (*n* = 4). Distinct letters within each graph represent statistically significant differences (*p* ≤ 0.05). Days (d).

Suc concentration was the highest with respect to all of the other detected sugars (Figure 2). Peach fruit kept at 20 °C displayed a constant Suc concentration throughout the postharvest period, except after 5 d of storage, where it decreased (Figure 2A). ‘Red Haven’ fruit stored at 0 °C exhibited a significant increase in Suc concentrations at 15 d of storage, displaying higher values than fruit stored at 5 °C, followed by a significant decrease at 30 d. However, fruit evaluated right after storage at 5 °C presented a significant and continuous decrease in Suc contents until 30 d (Figure 2A). From 3 d onwards, fruit kept at 5 °C exhibited significantly lower Suc concentrations than fruit stored at 0 °C. Fruit assessed after storage at 0 °C and 5 °C plus 3 d at 20 °C followed the same trends as fruit assessed directly out of cold storage, but displayed lower values (Figure 2A).

Glu and Fru concentration exhibited a significant and continuous increase throughout storage in fruit stored at 20 °C, reaching the highest values after 5 d (Figure 2B,C). Peach fruit immediately evaluated after storage at 0 °C maintained constant Glu and Fru concentrations throughout the assessed storage timepoints, while fruit exposed to 5 °C presented a significant increase in Glu and Fru from 15 d onwards, with significantly higher Glu and Fru concentrations than fruit stored at 0 °C (Figure 2B,C). Fruit sampled after storage at 0 °C and 5 °C plus 3 d at 20 °C followed the same trends, but exhibited higher values, with respect to fruit evaluated directly out of storage for each temperature, from 15 d onwards. Peach fruit stored for 30 d at 5 °C plus 3 d at 20 °C presented significantly higher Glu (Figure 2B) and Fru (Figure 2C) concentrations than fruit stored for 30 d at 0 °C plus 3 d at 20 °C.

The Sor concentration in fruit stored at 20 °C exhibited a significant decrease during storage (Figure 2D). In contrast, fruit directly assessed after storage at 0 °C and 5 °C presented a significant increase in Sor concentration, with the former and the latter reaching the highest concentrations at 15 d and 5 d, respectively, followed by a significant decrease afterwards, in both cases (Figure 2D). Fruit evaluated after storage at 0 °C and 5 °C plus 3 d at 20 °C followed similar trends with respect to fruit evaluated directly out of storage for each temperature, and particularly, fruit stored at 5 °C presented lower Sor contents after 3 d of storage at 20 °C from 15 d onwards (Figure 2D). Notably, from postharvest day 15 d onwards, ‘Red Haven’ fruit stored at 5 °C displayed significantly lower Sor contents than fruit kept at 0 °C, with fruit stored for 30 d at 5 °C followed by fruit stored for 3 d at 20 °C exhibiting the lowest Sor concentrations (Figure 2D).

### 2.3. Transcript Accumulation of Sugar Metabolism-Related Genes

**Figure 3:**
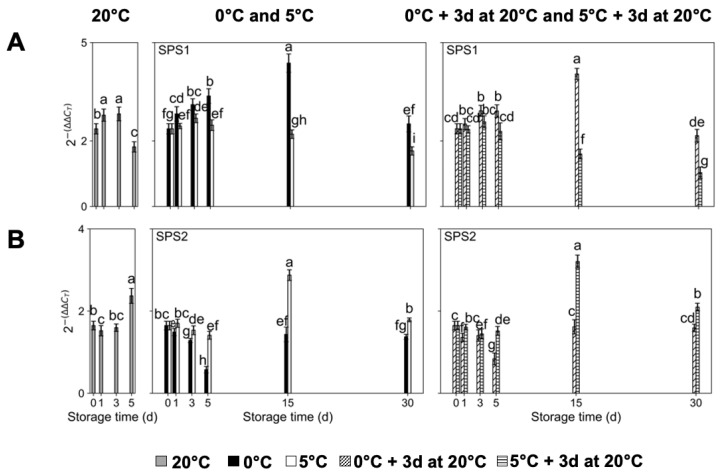
Transcript accumulation changes in sucrose biosynthesis-related genes in ‘Red Haven’ peaches during storage at different temperatures (20 °C, 0 °C, 5 °C, and 0 °C plus 3 d at 20 °C, and 5 °C plus 3 d at 20 °C). (**A**) *PpSPS1*; (**B**) *PpSPS2*. Values are means ± SE (*n* = 4). Distinct letters within each graph represent statistically significant differences (*p* ≤ 0.05). Sucrose phosphate synthase (SPS), days (d).

Regarding Suc synthesis-related genes, the transcript accumulation of sucrose phosphate synthase (*PpSPS1*) in fruit kept at 20 °C presented a significant decrease after 5 d of storage, displaying the lowest values at this timepoint (Figure 3A). ‘Red Haven’ fruit stored at 0 °C exhibited a continuous and significant increase in *PpSPS1* transcript accumulation until day 15, the timepoint at which it reached the highest value, and subsequently decreased at 30 d of storage. For fruit evaluated right after storage at 5 °C, *PpSPS1* expression levels were constant until day 5, after which they significantly decreased, although at all evaluation timepoints, they displayed significantly lower values than fruit stored at 0 °C (Figure 3A). Fruit exposed to storage for 3 d at 20 °C after storage at 0 °C and 5 °C presented the same trends as fruit assessed right out of cold storage (Figure 3A). From 5 d onwards, fruit kept at 5 °C exhibited significantly lower *PpSPS1* transcript accumulation than fruit stored at 0 °C, regardless of whether it was exposed for 3 d to a temperature of 20 °C, with fruit stored for 30 d at 5 °C plus 3 d at 20 °C displaying the lowest values (Figure 3A). In the case of *PpSPS2*, fruit stored at 20 °C presented a significant increase in expression levels after 5 d of storage, exhibiting the highest values at this timepoint (Figure 3B). The significantly lowest and highest *PpSPS2* transcript accumulation values amongst all evaluated cold-storage temperatures and timepoints were observed at 5 d for fruit stored at 0 °C, and at 15 d for fruit stored at 5 °C, respectively, independent of whether it was exposed for 3 d to a temperature of 20 °C (Figure 3B). For all evaluation timepoints, and independent of exposure for 3 d to a temperature of 20 °C, *PpSPS2* expression levels were higher in fruit assessed after storage at 5 °C than at 0 °C (Figure 3B).

**Figure 4:**
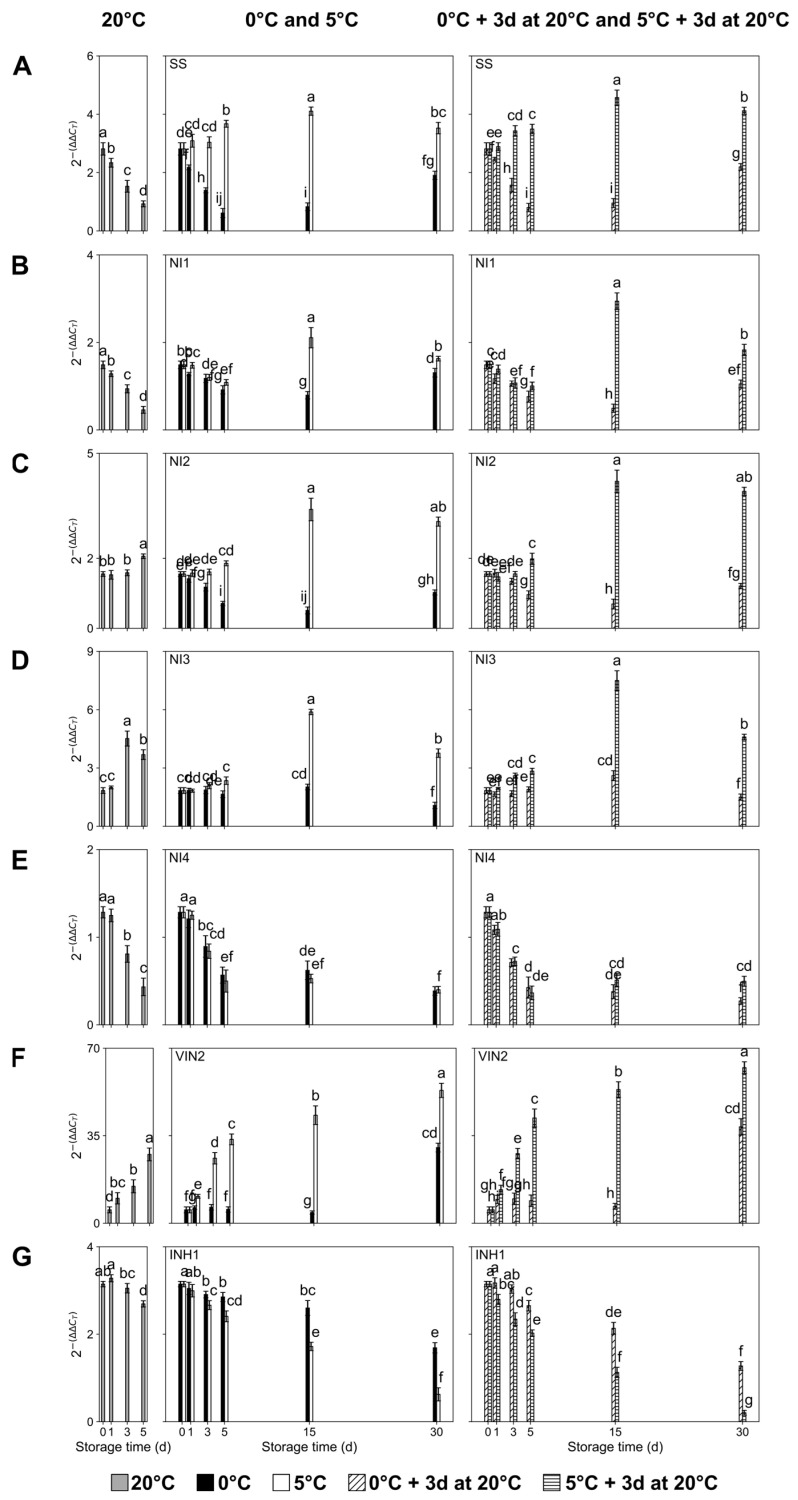
Transcript accumulation changes in sucrose breakdown-related genes in ‘Red Haven’ peaches during storage at different temperatures (20 °C, 0 °C, 5 °C, and 0 °C plus 3 d at 20 °C, and 5 °C plus 3 d at 20 °C). (**A**) *PpSS*; (**B**) *PpNI1*; (**C**) *PpNI2*; (**D**) *PpNI3*; (**E**) *PpNI4*; (**F**) *PpVIN2*; (**G**) *PpINH1*. Values are means ± SE (*n* = 4). Distinct letters within each graph represent statistically significant differences (*p* ≤ 0.05). Sucrose synthase (SS), neutral invertase (NI), vacuolar invertase (VIN), invertase inhibitor (INH), days (d).

Considering Suc, Glu, and Fru metabolism, and particularly Suc degradation-related genes, the expression levels of sucrose synthase (*PpSS*) significantly decreased in ‘Red Haven’ fruit kept at 20 °C throughout storage, as well as in fruit stored at 0 °C with and without exposure for 3 d to a temperature of 20 °C, although the latter displayed increased expression at 30 d of storage (Figure 4A). Contrariwise, peach fruit exposed to a temperature of 5 °C with and without exposure for 3 d to a temperature of 20 °C presented a significant increase in *PpSS* expression levels throughout storage, reaching the highest values at 15 d, followed by a significant decrease at 30 d (Figure 4A). For all evaluation timepoints, *PpSS* expression levels were significantly higher at 5 °C than at 0 °C, independent of whether fruits were exposed for 3 d to a temperature of 20 °C (Figure 4A).

In addition to *PpSS*, Suc can also be metabolized via invertases (neutral (*PpNI1*-*NI4)* and vacuolar (*PpVIN2*)). Regarding neutral invertases (*PpNI1*-*NI4*), fruit kept at 20 °C exhibited a significant decrease in the transcript accumulation of *PpNI1* (Figure 4B) and *PpNI4* (Figure 4E) throughout storage, while, inversely, *PpNI2* (Figure 4C) and *PpNI3* (Figure 4D) increased their expression. Peach fruit stored at 0 °C, independent of exposure for 3 d to a temperature of 20 °C, exhibited similar patterns of transcript accumulation for *PpNI1* (Figure 4B) and *PpNI2* (Figure 4C), showing a significant decrease throughout storage until day 15 followed by increased expression at 30 d; while, in the case of *PpNI3* (Figure 4D), the expression levels remained constant during the postharvest period and significantly decreased at 30 d, and for *PpNI4* (Figure 4E) there was a continuous and significant decrease until 30 d of storage. Fruit stored at 5 °C, independent of whether it was exposed for 3 d to a temperature of 20 °C, displayed the highest expression levels for *PpNI1* (Figure 4B), *PpNI2* (Figure 4C), and *PpNI3* (Figure 4D) at 15 d of storage, followed by a significant decrease at 30 d for *PpNI1* (Figure 4B) and *PpNI3* (Figure 4D), while the expression level of *PpNI4* (Figure 4E) significantly and continuously decreased throughout storage. In general, the transcript accumulation of *PpNI1* (Figure 4B) *PpNI2* (Figure 4C), and *PpNI3* (Figure 4D) was significantly higher in fruit stored at 5 °C compared to fruit stored at 0 °C, independent of whether it was exposed for 3 d to a temperature of 20 °C, from 5 d onwards. In the case of vacuolar invertase (*PpVIN2*), fruits kept at 20 °C, at 5 °C, and at 5 °C plus 3 d at 20 °C all presented significantly increased expression during storage (Figure 4F). Peach fruit assessed immediately after storage at 0 °C, as well as at 0 °C plus 3 d at 20 °C, exhibited low and constant expression values throughout the postharvest period, with a significant increase observed at 30 d (Figure 4F). Independent of whether it was exposed for 3 d to a temperature of 20 °C, fruit stored at 5 °C displayed significantly higher *PpVIN2* transcript accumulation than fruit stored at 0 °C, at all evaluation timepoints (Figure 4F). Inversely, the invertase inhibitor *PpINH1* (Figure 4G) displayed the opposite expression profile to *PpVIN2* (Figure 4F), with fruit stored at 5 °C displaying the significantly lowest transcript accumulation values at all evaluated timepoints compared to fruit stored at 0 °C, with and without exposure for 3 d to a temperature of 20 °C (Figure 4G). Furthermore, fruit stored at 20 °C presented a significant decrease in *PpINH1* transcript accumulation during the postharvest period (Figure 4G).

**Figure 5:**
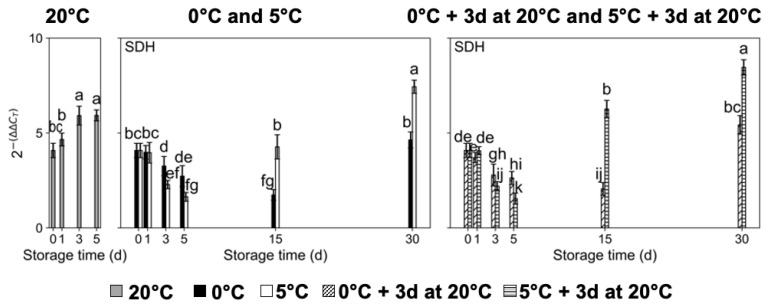
Transcript accumulation changes in sorbitol dehydrogenase (*PpSDH*), a sorbitol breakdown-related gene, in ‘Red Haven’ peaches during storage at different temperatures (20 °C, 0 °C, 5 °C, and 0 °C plus 3 d at 20 °C, and 5 °C plus 3 d at 20 °C). Values are means ± SE (*n* = 4). Distinct letters within each graph represent statistically significant differences (*p* ≤ 0.05). Days (d).

With respect to Sor breakdown-related genes, sorbitol dehydrogenase (*PpSDH*) expression levels significantly increased in ‘Red Haven’ fruit kept at 20 °C, displaying the significantly highest transcript accumulation for *PpSDH* at 3 d and 5 d of storage (Figure 5). Peach fruit stored at 0 °C with and without exposure for 3 d to a temperature of 20 °C exhibited a significant decrease in *PpSDH* expression levels until 15 d of storage, and then increased after 30 d (Figure 5), while in fruit exposed to a temperature of 5 °C, *PpSDH* transcript accumulation decreased until postharvest day 5 and significantly increased at 15 d and 30 d of storage. In fact, fruit stored at 5 °C with and without exposure for 3 d to a temperature of 20 °C presented significantly higher expression levels for *PpSDH* at 15 d and 30 d of storage than fruit stored at 0 °C (Figure 5).

### 2.4. Associations Amongst Chilling Injury Incidence, Sugar Concentration, and Sugar Metabolism-Related Genes

**Figure 6:**
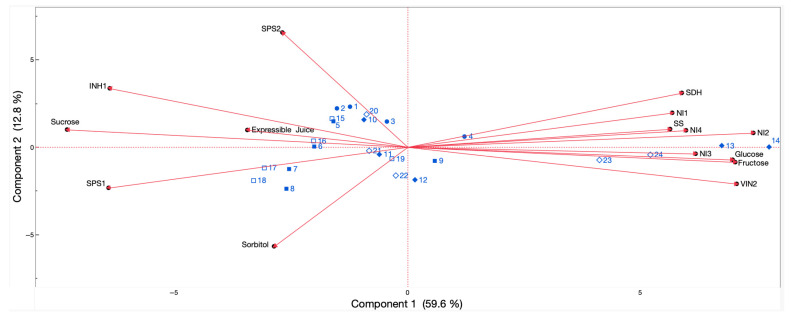
Principal component analysis depicting expressible juice contents, sugar concentration, and transcript accumulation of sugar metabolism-related genes in ‘Red Haven’ peaches during postharvest storage at different temperatures and followed by exposure for 3 d to a temperature of 20 °C. Numbers accompanied by different symbols depict the diverse temperature-storage time treatments used in this study (•1 (fruit stored at 20 °C for 0 d), •2 (fruit stored at 20 °C for 1 d), •3 (fruit stored at 20 °C for 3 d), •4 (fruit stored at 20 °C for 5 d), ▪5 (fruit stored at 0 °C for 1 d + 3 d at 20 °C), ▪6 (fruit stored at 0 °C for 3 d + 3 d at 20 °C), ▪7 (fruit stored at 0 °C for 5 d + 3 d at 20 °C), ▪8 (fruit stored at 0 °C for 15 d + 3 d at 20 °C), ▪9 (fruit stored at 0 °C for 30 d + 3 d at 20 °C), ♦10 (fruit stored at 5 °C for 1 d + 3 d at 20 °C), ♦11 fruit stored at (5 °C for 3 d + 3 d at 20 °C), ♦12 (fruit stored at 5 °C for 5 d + 3 d at 20 °C), ♦13 (fruit stored at 5 °C for 15 d + 3 d at 20 °C), ♦14 (fruit stored at 5 °C for 30 d + 3 d at 20 °C), □15 (fruit stored at 0 °C for 1 d), □16 (fruit stored at 0 °C for 3 d), □17 (fruit stored at 0 °C for 5 d), □18 (fruit stored at 0 °C for 15 d), □19 (fruit stored at 0 °C for 30 d), ◊20 (fruit stored at 5 °C for 1 d), ◊21 (fruit stored at 5 °C for 3 d), ◊22 (fruit stored at 5 °C for 5 d), ◊23 (fruit stored at 5 °C for 15 d), ◊24 (fruit stored at 5 °C for 30 d)). The gene codes used match those shown in Figure 3, Figure 4 and Figure 5.

The results from the PCA indicated that explanation of the total variation (72.4%) was achieved via principal component 1 (59.6%) and principal component 2 (12.8%) (Figure 6). Throughout principal component 1, the distribution of the different temperature and storage time treatments was defined by Suc content, as well as by the expression levels of *PpSPS1* and *PpINH1* on the negative side of the axis, while it was defined by Glu and Fru contents, as well as by the expression levels of *PpVIN2* and *PpNI2*, on the positive side of the axis (Figure 6). Fruit that was evaluated right after 0 °C and 5 °C storage was grouped on the negative side of the first principal component until 30 d and up to 5 d of storage, respectively, while fruits exposed to temperatures of 0 °C and 5 °C plus 3 d at 20 °C were also located on the negative side of the first principal component until up to 15 d and 3 d of storage, respectively. Likewise, fruit kept at 20 °C was positioned on the negative side of principal component 1 until 3 d of storage. As the postharvest timepoints increased, fruit stored at 20 °C for 5 d, and fruit stored at 0 °C plus 3 d at 20 °C at 30 d, and at 5 °C plus 3 d at 20 °C at 5 d, transitioned towards the positive side of principal component 1, displaying midway positioning. Moreover, fruits kept at 5 °C with and without exposure for 3 d to a temperature of 20 °C and assessed after 15 d and 30 d of storage were placed on the positive side of the first principal component, with the former presenting a more positive placement on the axis than the later (Figure 6). Throughout principal component 2, the distribution of the different temperature and storage time treatments was defined by Sor content on the negative side of the axis, while it was defined by the expression levels of *PpSPS2* on the positive side of the axis (Figure 6).

In this study, expressible juice content was significantly and positively correlated with the transcript accumulation of *PpINH1* (*r* = 0.64) and Suc concentration (*r* = 0.56), while the former and the later were also positively associated between them (*r* = 0.81). Suc concentration and the transcript accumulation of *PpINH1* were also positively correlated with the expression levels of *PpSPS1* (*r* = 0.85 and *r* = 0.67, respectively), but negatively associated with Glu and Fru contents (*r* ≤ −0.81), as well as to the transcript accumulation of Suc degradation-related genes *PpVIN2* (*r* ≤ −0.89), *PpNI1* to *PpNI4* (*r* ≤ −0.57), and *PpSS* (*r* ≤ −0.61). *PpINH1* expression levels were also positively correlated with *PpSPS2* (*r* = 0.67). Additionally, Glu and Fru concentrations were significantly and positively correlated between them (*r* = 0.99), as well as with all Suc breakdown-related genes (*r* ≥ 0.52), while they were negatively associated with the transcript accumulation of *PpSPS1* (*r* ≤ −0.64). All Suc degradation-related genes were also positively correlated amongst them (*r* ≥ 0.58).

Sor concentration was significantly and negatively correlated with the transcript accumulation of *PpSDH* (*r* = −0.82) and positively associated with the expression levels of *PpSPS1* (*r* = 0.60). The transcript accumulation of *PpSDH* showed a significantly positive correlation with Glu and Fru contents (*r* ≥ 0.77) as well as with the expression levels of all the Suc degradation-related genes (*r* ≥ 0.55) while showing negative correlations with Suc concentration (*r* = −0.64) and the transcript accumulation of *PpSPS1* (*r* = −0.72) and *PpINH1* (*r* = −0.59).

## 3. Discussion

**Figure 7:**
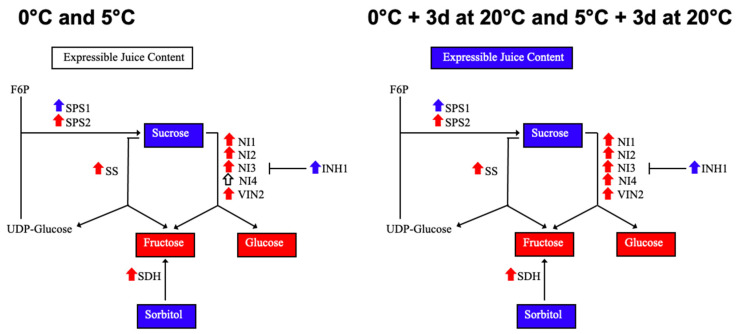
Summary of alterations in sugar metabolism and expressible juice content that modulate changes in ‘Red Haven’ peach chilling injury susceptibility 30 d postharvest at 0 °C and 5 °C, with and without exposure for 3 d to temperature of 20 °C. Expressible juice content and sugars are shown in blue and red rectangles that denote that their concentration was higher after storage at 0 °C or 5 °C, respectively. White rectangles indicate lack of differences between storage at 0 °C or 5 °C. Blue and red arrows facing up indicate gene transcript accumulation that was induced after storage at 0 °C and 5 °C, respectively. White arrows denote lack of differences between storage at 0 °C and 5 °C. Fructose-6-Phosphate (F6P); sucrose phosphate synthase (SPS); sucrose synthase (SS); neutral invertase (NI); vacuolar invertase (VIN); invertase inhibitor (INH); sorbitol dehydrogenase (SDH).

We conducted an examination of the responses to chilling injury incidence and sugar metabolism-related gene and metabolite changes throughout the postharvest period of ‘Red Haven’ peach fruit exposed to temperatures of 0 °C and 5 °C during storage, followed or not by exposure for 3 d to a temperature of 20 °C (to simulate retail shelf conditions), together with fruit stored at 20 °C. Additionally, significant associations between peach chilling injury development and sugar metabolism were identified. This work not only shows that changes in sugar metabolism under different storage temperatures start occurring during cold storage, but also demonstrates that these modifications are further maintained after exposure to simulated retail shelf conditions (3 d at 20 °C), priming ‘Red Haven’ fruit to cope or not with chilling stress during postharvest. The results from this study are summarized in Figure 7.

Proper peach fruit ripening and a lack of chilling injury development, particularly through the symptom of mealiness, are linked to high expressible juice contents, while conversely, a decline in expressible juice contents indicates the presence of mealiness in the fruit [38]. Previous studies have shown that peaches displaying less than 40% expressible juice contents are classified as mealy, and thus present chilling injury incidence [39]. In this study, ‘Red Haven’ fruit stored for 30 d at 5 °C plus fruit stored for 3 d at 20 °C, exhibited chilling injury incidence, which is in agreement with previous research on this cultivar [40], as the expression of the disorder develops once the fruit is transferred from storage to ambient conditions (20 °C), instead of when it is directly assessed after cold storage [6]. A lack of juiciness is known to be directly related to alterations in the fruit’s textural properties, such as fruit softening, which is, in turn, influenced by alterations in fruit turgor pressure and cell wall metabolism [8,41,42]. Juicy fruits are capable of preserving their capacity to induce the synthesis and trigger the action of several cell wall-modifying enzymes throughout cold storage as well as during exposure for 3 d to a temperature of 20 °C, avoiding the occurrence of alterations in pectin metabolism induced by cold, while mealy fruits are not [5,8,29,43,44]. Although ‘Red Haven’ is known to be a freestone, melting-flesh cultivar presenting a quick fruit softening capacity [45,46], the incidence of mealiness in fruit stored for 30 d at 5 °C, followed by exposure for 3 d to a temperature of 20 °C, emphasizes the critical role that storage temperature plays on chilling injury disorder development.

Sugars, in addition to being significant sources of carbon and energy as well as contributors to fruit flavor, are key players in alleviating abiotic stresses in plants, mainly due to their ability to work as osmoprotectants and function as cell membrane stabilizers and as cryoprotectants, as well as their capacity for reactive oxygen species (ROS) scavenging [18,20,21,22,23,24]. In peach fruit, although the main translocated assimilates are Suc and Sor, Suc is the predominant sugar in ripe fruit, with smaller concentrations of Glu, Fru, and Sor [1,27], consistent with the results of this work. The significant and continuous decrease in Suc accumulation in fruit stored at 5 °C observed during cold storage and maintained after exposure for 3 d to a temperature of 20 °C, in contrast to the increased Suc concentration observed for fruit stored at 0 °C throughout most of the storage timepoints (with and without exposure for 3 d to a temperature of 20 °C), could play a crucial role in chilling injury development in the former. The maintenance of the significantly higher Suc concentration from 15 d onwards in fruit stored at 0 °C in comparison to fruit stored at 5 °C after fruits were transferred storage at 20 °C for 3 d indicates a potential effect of Suc in decreasing chilling injury expression, both during cold storage as well during subsequent exposure to simulated retail shelf conditions (3 d at 20 °C). Although previous studies in peaches and nectarines [16,17,33,47,48,49], as well as apricots [50], that assessed fruit immediately after cold storage also associated increased Suc concentration with enhanced chilling injury tolerance, the present work is unique in demonstrating that this trend is maintained after exposure for 3 d to a temperature of 20 °C. Furthermore, in tomato fruit, chilling tolerance enhanced Suc concentration during postharvest storage [51], while in alfalfa roots [52] and grape branches [53], Suc was significantly accumulated during the chilling acclimation process. Likewise, hot air treatment in mandarins [54] and peaches [3] prevented a decline in Suc concentration during cold storage, which directly contributed to decreased chilling injury incidence in these fruit. As oxidative stress has been reported to be a significant contributor to chilling damage [28], the capability of Suc for enhancing chilling injury protection in fruit may be due to its strong antioxidant properties, which are known to be higher than those of Glu and Fru [55]. In fact, Suc hydroxyl scavenging capacity has been shown to have equal effectiveness to gluthatione, a conventional antioxidant [25]. Additionally, Suc seems to play an important role in membrane structure protection by preventing the leakage and degradation of proteins, boosting its capability to resist chilling injury [16,56]. The positive correlation obtained in this study between Suc concentration and expressible juice supports the idea that increased Suc accumulation is critical for reducing the symptom of mealiness and, thus, chilling injury incidence in ‘Red Haven’ peaches during cold storage, as well as after exposure to simulated retail shelf conditions (3 d at 20 °C). In fruit stored at 20 °C, the fact that the constant Suc concentration throughout the postharvest period only decreased after 5 d of storage could imply that Suc breakdown occurs only in the later stages of the ripening process, as it has been previously shown that during the early stages of ripening, it is Sor that can be readily used as carbon fuel [12,57], supporting this study’s results.

The hexoses Glu and Fru presented very similar concentration patterns throughout all of the assessed storage temperatures, indicating their positive correlation. However, there are discrepancies among different studies regarding the role of hexoses in chilling injury tolerance. On one hand, several studies have reported that increased Glu and Fru concentrations throughout the postharvest period can contribute to reduce chilling injury incidence in different fruits, such as loquat [15,58] and apricot [30], as Glu has been related to the synthesis of antioxidant compounds as well as to serving as a substrate for ROS scavenging-related metabolic pathways [29], while Fru has been shown to confer a higher antioxidant capacity than Glu [59], thus playing an important role in the adaptation of plants to cold-induced oxidative stress. On the other hand, in non-melting peaches [28] and papaya [60], some authors have demonstrated that high concentrations of Suc, as well of the hexoses Glu and Fru, were associated with a decreased incidence of chilling injury. Nevertheless, in this work, there were no changes in Glu and Fru accumulation in fruit stored at 0 °C, but there was a significant increase in hexose concentrations in fruit stored at 5 °C (with and without exposure for 3 d to a temperature of 20 °C) from 15 d onwards, with the highest Glu and Fru accumulation occurring in chilling-injured fruit. The negative correlation between hexoses and expressible juice contents obtained here implies that hexoses are not related to chilling injury tolerance in ‘Red Haven’ peaches. These discrepancies could be due to differences in the relationship between sugar metabolism and chilling injury tolerance between fruit species as well as within the same fruit species. Between fruit species, the inconsistent results could be explained by loquats being a non-climacteric fruit, as well as by the study targeting the evaluation of a red-fleshed cultivar and the effect of heat-induced chilling tolerance, which differ from the conditions assessed in the current work, while in the case of apricots, the study indicated that internal browning was the only expressed chilling injury symptom, which was not observed in ‘Red Haven’. Within the same fruit species, the discrepancy in the results between the non-melting peach study and the present ‘Red Haven’ study could be explained by the non-climacteric behaviour as well as the lack of detection of the chilling injury symptom of mealiness in the former, which differ from the later. Nevertheless, the results from the present work are in agreement with studies on other melting peach cultivars showing that Suc is more effective in contributing to decreased chilling injury incidence than Glu and/or Fru [16,17]. This could be explained by the higher metabolic accessibility to respiratory loss that Glu and Fru have when compared to Suc [23,61,62], and consequently, the maintenance of higher Suc concentrations in peach may balance energy savings and respiration and therefore improve chilling injury tolerance in ‘Red Haven’. Suc has also been shown to be more effective in protecting membranes than Glu and Fru in peach fruit, and this study indicates that this not only happens during cold storage but is also maintained after exposure to simulated retail shelf conditions (3 d at 20 °C). Furthermore, as the ripening process comprises a series of oxidative reactions [63,64], i.e., during fruit softening [65], this could explain the continuous increase in Glu and Fru concentrations in ‘Red Haven’ fruit stored at 20 °C throughout the postharvest period. This is in agreement with previous studies [12] and is supported by the negative correlation between Suc and hexoses in this work, as the latter result from cleavage of the former.

The concentrations of Suc and the sugar alcohol Sor have been shown to differ considerably among Rosaceae family members, with peaches presenting higher Suc and lower Sor abundance [66], conversely to cherries [67] or plums [23,68]. However, together with Suc, Sor accumulation has also been associated with cold stress amelioration in non-melting peaches [69] and mangoes [70,71]. In this study, the lack of significant positive correlations between Sor and expressible juice contents could be explained by the potential role of Sor in decreasing chilling injury incidence in the early stages of the disorder’s development, but this needs to be further investigated. This is supported by ‘Red Haven’ fruit stored at 5 °C (with and without exposure for 3 d to a temperature of 20 °C) exhibiting the highest Sor accumulation amongst all treatments after 5 d of storage, and then significantly decreasing from 15 d onwards, while this only happened after 30 d in fruit stored at 0 °C (with and without exposure for 3 d to a temperature of 20 °C). Decreased Sor accumulation was also observed in the early stages of chilling-injured ‘Spring Lady’ [29], ‘Yulu’ [16], and ‘Jinqiuhongmi’ [1] peaches assessed immediately after cold storage, although the present work demonstrates that in the case of ‘Red Haven’, this is also maintained after simulated retail shelf conditions (3 d at 20 °C). The exposure of peach fruit to cold temperatures can activate the accumulation of cryoprotectants, such as Sor, as a result of the cold acclimation process, which helps maintain the stability of phospholipid membranes via the regulation of osmotic balance [33]. The capacity of Sor to decrease chilling injury incidence might be a result of its osmoprotective function [72], which promotes oxidative damage reduction by stabilizing membranes and preventing structural deterioration during postharvest cold storage [70,73], which is then maintained during simulated retail shelf conditions (3 d at 20 °C). Furthermore, the significant and continuous decrease in Sor concentration in ‘Red Haven’ fruit stored at 20 °C is consistent with previous studies [12,74] indicating that Sor does not accumulate in peach fruit after harvest as it is rapidly broken down into Glu and Fru [75].

The transcript accumulation of sugar metabolism-related genes allowed for a deeper understanding of the observed changes in Suc, Glu, Fru, and Sor concentrations occurring throughout storage at the different temperatures assessed in this study. Regarding Suc, Glu, and Fru metabolism, SPS encodes the key Suc biosynthesis enzyme [31], while invertases as well as SS are responsible for breaking down Suc into hexoses [14,76]. Additionally, invertase inhibitors play important roles regulating invertases [33]. The decrease in the expression of *PpSPS1*, together with the increased transcript accumulation of *PpSS*, neutral invertases *PpNI1*, *PpNI2*, *PpNI3*, and vacuolar invertase *PpVIN2*, throughout the postharvest period, together with the significantly lowest *PpINH1* expression levels at all evaluated timepoints in fruit stored at 5 °C compared to fruit stored at 0 °C, can contribute to explaining the continuous decrease in Suc, as well as the continuous increase in Glu and Fru concentration observed in the former, thereby impairing tolerance to chilling injury development (Figure 7). The transcript changes described above occur in ‘Red Haven’ fruit assessed immediately after cold storage and are maintained after exposure to simulated retail shelf conditions (3 d at 20 °C), although in the latter, *PpNI4* gene expression is additionally higher in fruit that was stored at 5 °C compared to fruit stored at 0 °C (Figure 7).

While fruit stored at 5 °C displayed a higher transcript accumulation of *PpSPS2* when compared to fruit stored at 0 °C, immediately after cold storage as well as after exposure for 3 d to a temperature of 20 °C, the lower Suc accumulation in fruit exposed to 5 °C can be attributed partly to the inability of *PpSPS2* to overcome the increased Suc breakdown-related gene expression exhibited at this temperature. Furthermore, and consistent with this study’s results, it was reported that the upregulation of *PpSPS1* was critical to avoiding the disappearance of Suc during chilling temperature storage in Rosaceae fruit such as peach [1,16,17] and Japanese pear [77] when these fruits were assessed immediately after cold storage, although the current work additionally demonstrates that this is also the case after exposure for 3 d to a temperature of 20 °C in ‘Red Haven’. The significantly positive correlation displayed by the transcript accumulation of *PpSPS1* and Suc concentration, in contrast to the lack of association between *PpSPS2* and Suc concentration as well as between both SPS genes assessed in this study, supports the above. Regarding invertases, the significantly higher transcript accumulation of the neutral invertases *PpNI1*, *PpNI2*, and *PpNI3* observed during most of the assessed postharvest timepoints in fruit evaluated directly after storage at 5 °C, in comparison to fruit evaluated after storage at 0 °C, is in agreement with previous research on peach [16,17]. Notably, in the present study, these differences were shown to be maintained even after exposure for 3 d to a temperature of 20 °C, and additionally accompanied by a higher transcript accumulation of *PpNI4* in ‘Red Haven’ fruit stored at 5 °C in comparison to fruit stored at 0 °C after exposure to simulated retail shelf conditions, indicating increased Suc breakdown into hexoses in fruit stored at 5 °C. Interestingly, fruit kept at 20 °C only displayed an increased expression level throughout ripening for *PpNI2* and *PpNI3*, consistent with the results obtained for ‘Dixiland’ peaches [12,57] and suggesting a difference in the promotion of particular neutral invertase transcripts with and without cold storage exposure. Nonetheless, the decreased transcript accumulation of *PpNIN4* throughout the postharvest period for all storage temperatures assessed in this work suggests that *PpNIN4* does not play a role during peach fruit ripening, which could be compensated for by the induction of the other NIs, although this requires further analyses. Moreover, in the case of acid invertases, they are considered the most important enzymes in fruit Suc metabolism [35,78]. Particularly in peach, only vacuolar invertase *PpVIN2* expression has been shown to be sensitive to chilling temperatures [36], which explains the positive and negative correlations of *PpVIN2* with hexoses and Suc, respectively, in this study. Furthermore, VIN2 activity is inhibited by the action of the invertase inhibitor INH1 [33], explaining the significant negative association obtained between *PpVIN2* and *PpNINH1* in this work. The increasing transcript accumulation of *PpVIN2*, together with the decreasing expression of *PpNINH* throughout the postharvest ripening of ‘Red Haven’ fruit, which was statistically more prominent in fruit stored at stored at 5 °C than at 0 °C, contributes to the significant reduction in Suc and increased hexose concentration at this temperature. On the other hand, the repression of *PpVIN2* transcript levels by *PpNINH1*—which was also observed in previous studies on peaches assessed directly after cold storage [1,33,79,80], and which, in this study, for the first time, was also shown to be maintained after exposure for 3 d to a temperature of 20 °C—explains the conservation of higher Suc levels during storage in fruit kept at 0 °C compared to fruit kept at 5 °C, emphasizing the crucial role acid invertases play in peach fruit sugar composition. The importance of the latter has also been previously reported in Japanese pear [77], loquats [15], and potato tubers [81] assessed immediately after cold storage.

Concerning Sor metabolism, Sor breakdown into Fru is catalyzed by *PpSDH* [82], supporting the significantly negative correlation obtained in this work between Sor and *PpSDH*, in agreement with earlier reports in peaches [69] and plums [23,83] during storage. The increased transcript accumulation of *PpSDH* in fruit stored at 5 °C, observed in fruit assessed immediately after cold storage and maintained after exposure for 3 d to a temperature of 20 °C, particularly after 15 d of storage, can explain the significantly lower Sor concentrations displayed by these fruits compared to fruit stored at 0 °C from this timepoint onwards. The significantly highest expression of *PpSDH* after 3 and 5 d of postharvest ripening at 20 °C supports the observed increased Sor catabolism and Fru accumulation in this fruit, consistent with previous research on peaches [84].

The placement of the different temperature treatments and storage timepoints throughout principal component 1 of the PCA suggests that tolerance to chilling injury in ‘Red Haven’ peaches is primarily characterized by increased Suc concentration combined with the transcript accumulation of genes which promote Suc biosynthesis and decrease Suc catabolism, as well as by the presence of Sor. The latter is associated with fruit assessed right after storage at 0 °C (after 30 d of storage), with fruit exposed to a temperature of 0 °C plus 3 d at 20 °C (after 15 d of storage), and with fruit kept only at 20 °C. Conversely, susceptibility to chilling injury is characterized by increased Glu and Fru concentrations in parallel with the transcript accumulation of genes which promote Suc and Sor catabolism and, thus, Glu and Fru biosynthesis. The latter is related to fruit kept at 5 °C, with and without exposure for 3 d to a temperature of 20 °C, from 5 d to 15 d of storage onwards, respectively. Studies which incorporate a diverse set of peach cultivars and a wider range of storage temperatures are underway to assess the consistency and expand upon the results of this work.

## 4. Materials and Methods

### 4.1. Fruit Material

The current study builds on earlier research [2], and as such, the same fruit samples and postharvest storage treatments as previously described were used. A commercial orchard situated in Westminster, Maryland, United States, was used for harvesting fruit from the mid-season freestone, yellow, and melting-flesh juicy and sweet peach (*Prunus persica* (L.) Batsch) cv. ‘Red Haven’ in the 2020 season. Fruits were collected at optimal maturity, corresponding to a change in external ground color from green to yellow and to a flesh firmness of 55–65 N, as defined previously [2].

### 4.2. Postharvest Fruit Storage

Postharvest fruit storage treatments were conducted following a method that was previously described [2]. Fruits were separated randomly into three different groups after harvest, and each group was stored at a different temperature (0 °C, 5 °C, and 20 °C). Fruits were assessed at harvest (0 d) and at 1, 3, 5, 15, and 30 d of storage, followed or not by storage for 3 d at 20 °C (to simulate retail shelf conditions). Four biological replications were evaluated for each temperature and storage timepoint. Each replication comprised four fruits used to analyze the expressible juice content, and then their skin was removed, and they were cut into pieces, frozen, and homogenized in liquid nitrogen and kept at −80 °C for downstream analyses.

### 4.3. Chilling Injury Determination

An assessment of expressible juice was conducted to measure the development of the symptom of mealiness, and therefore CI determination, by following a method that was previously described [9,38].

### 4.4. Sugar Quantification

Sugar quantification in peach flesh tissue was executed using a previously described methodology [83,85] via high-performance liquid chromatography (HPLC) (Agilent HPLC 1100 series, Palo Alto, Santa Clara, CA, USA) with a refractive index detector (RID).

### 4.5. Real-Time Quantitative RT-PCR Analysis

RNA was extracted from peach fruit flesh using a method outlined in earlier reports [83,86]. First-strand complementary DNA synthesis and real-time quantitative PCR were carried out following previously described methods [83]. The full list of primer sets used for amplifying the target genes in this study is given in Table S1 and was designed based on previously published primer sequences [16,33]. The Comparative Cycle Threshold Method [87] was used for the analysis of relative gene expression. The normalization and calibration of transcript values were conducted relative to the expression of the reference gene Translation Elongation Factor 2 (*PpTEF2*) [88].

### 4.6. Statistical Analysis

A completely randomized design was used in this study. The four biological replication sample means were analyzed via two-way analysis of variance (ANOVA) using Tukey’s HSD test to compare between postharvest assessment timepoints (0, 1, 3, 5, 15 and 30 d) and temperatures (comparisons between fruits stored at 0 °C and 5 °C were conducted independently of comparisons between fruits exposed to a temperature of 0 °C + 3 d at 20 °C and a temperature of 5 °C + 3 d at 20 °C) for the features of expressible juice, sugar quantification, and relative gene expression (*p* ≤ 0.05). Additionally, for the same features, the four biological replication sample means were analyzed via one-way analysis of variance (ANOVA) using Tukey’s HSD test to compare between postharvest assessment timepoints for fruit kept at 20 °C.

Pearson’s correlation coefficients (r) were obtained for each paired combination of the measured features, using mean-centered data at a α = 0.05 significance level. Principal component analysis (PCA), conducted using data standarization, denoted the relationships between the measured features (expressible juice, sugar quantification, relative gene expression values) and the examined postharvest assessment timepoints and temperatures via a ‘biplot’ graph. A Scree test was applied to determine how many principal components were needed to capture the majority of the data variation. The data were analyzed statistically using JMP software (version 15.2, SAS Institute, Cary, NC, USA).

## 5. Conclusions

‘Red Haven’ fruit stored at 5 °C plus 3 d at 20 °C exhibited chilling injury incidence via the symptom of mealiness after 15 d of storage. Chilling injured fruit was associated primarily with reduced Suc, but increased Glu and Fru concentration, while Sor contents were also lower. This was accompanied by the decreased transcript accumulation of Suc biosynthesis-related genes and the increased expression of Suc and Sor catabolism-related genes. Sugar metabolism changes that occurred during cold storage were maintained after exposure to simulated retail shelf conditions (3 d at 20 °C). This work suggests that storage at 0 °C better preserves sucrose homeostasis, reducing chilling injury risk during cold storage as well as after exposure for 3 d to a temperature of 20 °C, and highlights the importance of sugar metabolites in priming fruit to cope with chilling stress.

## Data Availability

The data are contained in the article.

## Supplementary Materials

The following supporting information can be downloaded at: https://www.mdpi.com/article/10.3390/plants14142133/s1, Table S1: Primers used in qRT-PCR performed from RNA isolated from ‘Red Haven’ peach fruit flesh.
