# Supplementary material for: Understanding Chilling Injury and Sugar Metabolism-Related Genes and Metabolites in ‘Red Haven’ Peaches
Source: Plants (Basel). 2025 Jul 10;14(14):2133. doi: 10.3390/plants14142133 (PMC12298410; doi:10.3390/plants14142133)
Supplement: Supplementary file 1 [file plants-14-02133-s001.zip › plants-3714218-supplementary.pdf]

Supplementary Table S1. Primers used in qRT-PCR performed from RNA isolated from 'Red Haven' peach fruit flesh.

Gene name	Description	Primer orientation	Primer sequence (5' to 3')
PpSPS1	Sucrose phosphate synthase	Forward	TTGAGGCTACAGGAAAGGAAAG
		Reverse	GGACGCTCCTCTGAATGAATAG
PpSPS2	Sucrose phosphate synthase	Forward	CTTCCCTTTGTGGTGGATTTAG
		Reverse	GAGTTCCTTAACAGGGGGAATC
PpSS	Sucrose synthase	Forward	ATGAGGAGAAGGCTGAGATGAAG
		Reverse	CAAGTAGCGAATGTTGGAAGTC
PpNI1	Neutral invertase	Forward	TGCTCTGGAGTATGAAGAATGG
		Reverse	ATCCACTGCCTTTTGTGCTAAC
PpNI2	Neutral invertase	Forward	CTATGACACCAAAAGGGGTAGG
		Reverse	GCTTTCTTCTTGGGTTAGCACT
PpNI3	Neutral invertase	Forward	GGGTACTCCAAAGCAAAATGAC
		Reverse	TATGACCAAGGGGTATTCTTCG
PpNI4	Neutral invertase	Forward	ATGGTATGGCGATTCTTTCATC
		Reverse	AGACCCTCAGTTGTGGTAGCTC
PpINH1	Invertase inhibitor	Forward	ATGTCCCACAAGGCAGTCAA
		Reverse	CAGCCGCAACATCAAGAAGAG
PpVIN2	Vacuolar invertase	Forward	ACAAGGTCTTCCGTGGCAAA
		Reverse	AGCAGCCCCATAAATTGCCT
PpSDH	Sorbitol dehydrogenase	Forward	GCAGACTTTGTTGTTCAAGAGC
		Reverse	TCATGTCAGGGCAGAGATTG
PpTEF2	Translation elongation factor	Forward	TGAAGGAGAGGGAAGGTGAAAG
		Reverse	GGTGTGACGATGAAGAGTGATG
